# Polymorphic Sites at the Immunoregulatory CTLA-4 Gene Are Associated with Chronic Chagas Disease and Its Clinical Manifestations

**DOI:** 10.1371/journal.pone.0078367

**Published:** 2013-10-24

**Authors:** Fabrício C. Dias, Tiago da S. Medina, Celso T. Mendes-Junior, Roberto O. Dantas, Cristina W. Pissetti, Virmondes Rodrigues Junior, Renata Dellalibera-Joviliano, José A. Marin-Neto, Fredy R. S. Gutierrez, Philippe Moreau, João S. Silva, Eduardo A. Donadi

**Affiliations:** 1 Departamento de Bioquímica e Imunologia, Faculdade de Medicina de Ribeirão Preto, Universidade de São Paulo, Ribeirão Preto, São Paulo, Brazil; 2 Departamento de Química, Faculdade de Filosofia, Ciências e Letras de Ribeirão Preto, Universidade de São Paulo, Ribeirão Preto, São Paulo, Brazil; 3 Departamento de Clínica Médica, Faculdade de Medicina de Ribeirão Preto, Universidade de São Paulo, Ribeirão Preto, São Paulo, Brazil; 4 Laboratório de Imunologia, Universidade do Triângulo Mineiro, Uberaba, Minas Gerais, Brazil; 5 Departamento de Cirurgia e Anatomia, Faculdade de Medicina de Ribeirão Preto, Universidade de São Paulo, Ribeirão Preto, São Paulo, Brazil; 6 School of Medicine, University Antonio Nariño, Bogotá, Colombia; 7 CEA, Institute of Emerging Diseases and Innovative Therapies, Research Division in Hematology and Immunology, Saint-Louis Hospital, Paris, France; University of Texas Health Science Center San Antonio Texas, United States of America

## Abstract

**Background:**

Chagas disease affects approximately 10 million people mainly in Latin America. The immune regulation by the host seems to be an essential factor for disease evolution, and immune system inhibitory molecules such as CTLA-4 and PD-1 favor the maintenance of peripheral tolerance. Considering that polymorphisms at the immunoregulatory *CTLA-4* and *PDCD1* genes may alter their inhibitory function, we investigated the association of alleles, genotypes and haplotypes of polymorphic sites observed at the *CTLA-4* and *PDCD1* genes with different clinical manifestations of chronic Chagas disease (indeterminate, cardiac, digestive and mixed).

**Methods:**

The polymorphisms at the *CTLA-4* (-1722T/C, -318C/T and +49A/G) and *PDCD1* (*PD-1*.*3*G/A) genes were typed using TaqMan methodology in 277 chronic Chagas disease patients classified into four groups, according to clinical characteristics, and 326 non-infected controls.

**Results:**

Our results showed that *CTLA-4* -1722CC genotype (22%), -1722C allele (27%) and *CTLA-4* TCG (8.6%), TCA (26%) and CCA (15%) haplotypes were strongly associated with the indeterminate form, while the *CTLA-4* -318CT genotype (82%) and *CTLA-4* -318T allele (47%) were found mainly in patients with the mixed form of the disease. The *CTLA-4* TCG haplotype (10.2%) was associated with the digestive form. On the other hand, the *PD-1*.*3*G/A polymorphism was not associated with chronic Chagas disease and its clinical manifestations.

**Conclusions:**

Here, we showed that alleles, genotypes and haplotypes reported to increase the expression of the regulatory molecule CTLA-4 were associated with the indeterminate form of the disease. Taken together, our data support the idea that polymorphic sites at immunoregulatory genes may influence the development of Chagas disease variants.

## Introduction

The parasite *Trypanosoma cruzi* is the etiologic agent of Chagas disease, which affects mainly Latin American populations. An estimated population of 25 million is living at risk of infection, representing a prevalence of 10 million cases all over the world. It was estimated that this illness killed more than 10,000 people worldwide in 2008 [1]. Chagas disease presents acute and chronic phases. The acute phase, which lasts 1 to 2 months, is characterized by an asymptomatic period in most cases, although sometimes it may present acute myocarditis accompanied by cardiomegaly or meningoencephalitis, which can be lethal [2]. In the chronic phase, 60-70% of cases are asymptomatic and 30-40% of patients develop cardiac, digestive or cardiodigestive forms [3]. The symptoms of the cardiac form involve abnormalities of the heart conduction system, bradyarrhythmias and tachyarrhythmias, apical aneurysms, cardiac failure, thromboembolism, and sudden death [4-6]. The digestive manifestations are mainly megaesophagus and megacolon [7]; the main symptoms of megaesophagus are dysphagia with odynophagia, epigastric pain, regurgitation and ptyalism, while megacolon causes prolonged obstipation, abdominal distention, and large bowel obstruction due to fecaloma or sigmoid volvulus [3,8].

The resistance to *T. cruzi* infection is characterized by an increased production of INF-γ by CD4^+^ T cells, which leads to CD8^+^ T cell activation and differentiation [9]. However, CD8^+^ T cells are responsible for cytotoxicity against host infected cells leading to extensive fibrosis and cytolysis and contributing to heart damage [10]. The immune regulation by the host seems to be an essential factor for disease evolution, and inhibitory molecules such as CTLA-4 (Cytotoxic T Lymphocyte-Associated Antigen-4) and PD-1 (Programmed Death Receptor-1) favor the maintenance of peripheral tolerance, restraining T cell activation and proliferation.

The CTLA-4 molecule is expressed on activated T cell surface and the interaction of CTLA-4 with its ligands B7.1 and B7.2 generates a negative signal that regulates T cell activation and proliferation [11-13]. Moreover, regulatory T cells (Tregs) express high levels of CTLA-4 on their surface, indicating that this molecule may play an important role in their function [14]. The *CTLA-4* gene, located at chromosomal region 2q33, contains more than 100 polymorphic sites [15], and distinct polymorphisms have been associated with autoimmune and infectious diseases [16-20]. The CTLA-4 +49A/G single nucleotide polymorphism (SNP) is located in exon 1 and promotes a Threonine (A) to Alanine (G) substitution in the protein leader sequence at amino acid position 17 [21]. Threonine in this position results in a stronger interaction of CTLA-4 with B7.1 molecules, inducing a higher inhibitory effect on T-cell activation when compared to the presence of Alanine [22]. Moreover, the presence of Alanine at position 17 of the *CTLA-4* gene results in inefficient glycosylation and decreased molecule expression on the cell surface [23]. The *CTLA-4* -1722T/C and *CTLA-4* -318C/T SNPs were identified at the promoter region of the *CTLA-4* gene and some particular alleles may affect *CTLA-4* mRNA and protein levels [24,25].

The PD-1 molecule is expressed on the cell surface of activated T and B cells and myeloid lineage cells [26], and interacts with the ligands PD-L1 and PD-L2 [27,28]. Interaction of PD-1 with its ligands activates a cytoplasmic immunoreceptor tyrosine-based inhibitory motif (ITIM), inducing a negative signal that inhibits T and B cells activation and proliferation, leading to peripheral tolerance [28-30]. The PD-1 molecule is encoded by the *PDCD1* gene, which is located at chromosomal region 2q37.3 [31] and contains approximately 230 SNPs [32]. The *PD*-1.3G/A SNP is located at position +7146 at intron 4, and the presence of an A allele disrupts the binding affinity of the Runt-Related Transcription Factor (RUNX1/AML1), altering mRNA expression level, mRNA stability or both [31].

In this study, we investigated the association of alleles, genotypes and haplotypes at *CTLA-4* (-1722T/C rs733618, -318C/T rs5742909, +49A/G rs231775) and *PD*-1.3G/A (+7146 rs11568821) polymorphic sites with the diverse clinical manifestations of chronic Chagas disease. In summary, we observed that polymorphisms related to augmented expression of CTLA-4 molecule contributed to the development of the mild indeterminate chagasic form.

## Materials and Methods

### Ethics statement

The protocol was approved by the Institutional Review Board of the School of Medicine of Ribeirão Preto, University of São Paulo (Protocol number 11237/2009) and written informed consent was obtained from the patients.

### Subjects

A total of 277 chronic Chagas disease patients exhibiting positive serology for *T. cruzi* antigens were studied. Patients were classified into four groups according to clinical characteristics: cardiac, presenting or not congestive heart failure (n = 90), digestive (n = 67), mixed or cardiodigestive (n = 39) and indeterminate (asymptomatic) (n = 81). *T. cruzi*-infected patients were submitted to clinical examination, electrocardiography and chest, esophagus and colon contrast X-ray exams, thus classifying them into the cardiac, digestive, mixed or indeterminate form. Only patients exhibiting well defined clinical variants of Chagas disease were included in the study, and patients aged less than 18 years old and those exhibiting chronic infectious disorders were excluded. The total number of patients was determined after considering the exclusion criteria. Indeterminate patients exhibited this clinical variant for at least 20 years. A total of 326 unrelated healthy bone marrow donors of both sexes from the same geographic region of the patients exhibiting negative serology for *T. cruzi* antigens were randomly selected to constitute the control group. The demographic characteristics of patients and healthy controls are shown in Table 1.

**Table 1:** Demographic characteristics of patients with Chagas disease and healthy controls.

	**Clinical forms**					
**Characteristic**	**IND**	**CD**	**DG**	**MX**	**WG**	**HC**
n	81	90	67	39	277	326
Sex (Male/Female)	48/33	47/43	38/29	13/26	146/131	192/134
Age (Year)	55.0 ± 17.1	64.9 ± 15.3	61.2 ± 17.4	62.0 ± 15.3	60.7 ± 16.7	49.5 ± 13.8

IND (Indeterminate). CD (Cardiac). DG (Digestive). MX (Mixed). WG (Whole Group). HC (Healthy Control). Variables are expressed as mean ± SEM.

### DNA extraction and genotyping

Genomic DNA was extracted from peripheral blood leucocytes using a standard salting out procedure [33]. Genotyping of the *CTLA-4* -1722T/C (rs733618; Assay ID: C_2415791_10), *CTLA-4* -318C/T (rs5742909; Assay ID: C_27834180_10), CTLA-4 +49A/G (rs231775; Assay ID: C_2415786_20) and *PD*-1.3G/A (+7146) (rs11568821; Assay ID: C_57931290_10) single nucleotide polymorphisms was performed by real-time PCR using the TaqMan SNP (Single Nucleotide Polymorphism) Genotyping Assay and the StepOnePlus automatic instrument (Applied Biosystems, Foster City, CA), according to the manufacturer’s instructions.

### Statistical analysis

Allele and genotype frequencies were estimated by the direct counting method, and adherences of phenotypical proportions to expectations under Hardy-Weinberg equilibrium (HWE) were tested by the complete enumeration method using the GENEPOP 3.4 software [34]. Linkage Disequilibrium (LD) between *CTLA-4* SNPs was evaluated by means of Lewontin’s standardized coefficient *D’* and by a likelihood ratio test of linkage disequilibrium implemented at the ARLEQUIN software [35]. *CTLA-4* haplotype frequencies were estimated in each population sample by a coalescence-based method implemented in the PHASE v2 software [36]. The frequency of each allele, genotype or haplotype was compared between patients and controls by the two-sided Fisher exact test, with the aid of the GraphPad Instat 3.05 software, which was also used to estimate the Odds Ratio (OR) and its 95% Confidence Interval (CI). The Bonferroni correction was used to adjust the significance levels for multiple testing, resulting in α = 0.0011 (i.e., 0.05/44, where 44 indicates the number of tests involving 4 alleles in 11 group comparisons), α = 0.0004 (i.e., 0.05/132, where 132 indicates the number of tests involving 12 genotypes in 11 group comparisons), and α = 0.0006 (i.e., 0.05/88, where 88 indicates the number of tests involving 8 haplotypes in 11 group comparisons).

## Results

### Genotype and allele frequencies of CTLA-4 -1722T/C, -318C/T and +49A/G SNPs

To understand how polymorphic sites observed at genes related to immune regulation can be associated with clinical forms of Chagas disease, we firstly analyzed whether the frequencies of three polymorphisms at the *CTLA-4* gene were associated with clinical features. Genotype and allele frequencies of the *CTLA-4* gene polymorphisms in patients with chronic Chagas disease and controls are shown in Table 2. The -1722CC genotype frequency was increased in patients exhibiting the indeterminate form compared to controls (p = 0.0001), and also with patients presenting the cardiac (p<0.0001) and digestive (p<0.0001) variants. This genotype was not observed in patients with the cardiac, digestive and mixed forms. As a consequence, the indeterminate group exhibited an increased frequency of the -1722C allele frequency compared to controls (p<0.0001) and to patients with the cardiac (p<0.0001), digestive (p<0.0001) and mixed (p = 0.0001) forms.

**Table 2:** Genotype and allele frequencies of polymorphisms at the *CTLA-4* and *PDCD1* genes in patients with Chagas disease and healthy controls.

	**Clinical forms**												
	**IND**		**CD**		**DG**		**MX**			**WG**		**HC**	
**Polymorphism**	n	Freq.	n	Freq.	n	Freq.	n	Freq.		n	Freq.	n	Freq.
***CTLA-4* -1722**	(n = 69)		(n = 75)		(n = 64)		(n = 33)			(n = 241)		(n = 310)	
C/C	15	0.22*	0	0.00	0	0.00	0		0.00	15	0.06	18	0.06
C/T	7	0.10	7	0.09	6	0.09	3		0.09	23	0.10	23	0.07
T/T	47	0.68	68	0.91	58	0.91	30		0.91	203	0.84	269	0.87
C Allele	37	0.27*	7	0.05	6	0.05	3		0.05	53	0.11	59	0.10
T Allele	101	0.73*	143	0.95	122	0.95	63		0.95	429	0.89	561	0.90
***CTLA-4* -318**	(n = 70)		(n = 73)		(n = 64)		(n = 34)			(n = 241)		(n = 306)	
C/C	25	0.36	30	0.41	35	0.55	4		0.12*	94	0.39*	168	0.55
C/T	35	0.50	31	0.43	18	0.28	28		0.82*	112	0.47	119	0.39
T/T	10	0.14	12	0.16	11	0.17	2		0.06	35	0.14	19	0.06
C Allele	85	0.61	91	0.62	88	0.69	36		0.53*	300	0.62*	455	0.74
T Allele	55	0.39	55	0.38	40	0.31	32		0.47*	182	0.38*	157	0.26
**CTLA-4 +49**	(n = 77)		(n = 84)		(n = 67)		(n = 36)			(n = 264)		(n = 324)	
A/A	40	0.52	35	0.42	42	0.63	22		0.61	139	0.53	139	0.43
A/G	33	0.43	39	0.46	23	0.34	12		0.33	107	0.40	150	0.46
G/G	4	0.05	10	0.12	2	0.03	2		0.06	18	0.07	35	0.11
A Allele	113	0.73	109	0.65	107	0.80	56		0.78	385	0.73	428	0.66
G Allele	41	0.27	59	0.35	27	0.20	16		0.22	143	0.27	220	0.34
***PD-1.3***	(n = 81)		(n = 90)		(n = 66)		(n = 39)			(n = 276)		(n = 326)	
G/G	68	0.84	72	0.80	59	0.89	34		0.87	233	0.84	266	0.82
G/A	13	0.16	13	0.14	5	0.08	5		0.13	36	0.13	55	0.17
A/A	0	0.00	5	0.06	2	0.03	0		0.00	7	0.03	5	0.01
G Allele	149	0.92	157	0.87	123	0.93	73		0.94	502	0.91	587	0.90
A Allele	13	0.08	23	0.13	9	0.07	5		0.06	50	0.09	65	0.10

IND (Indeterminate). CD (Cardiac). DG (Digestive). MX (Mixed). WG (Whole Group). HC (Healthy Control).

* frequencies that show statistical differences.

The -1722T allele frequency was decreased in the indeterminate group compared to controls (p<0.0001) and to patients with the cardiac (p<0.0001), digestive (p<0.0001) and mixed (p = 0.0001) forms.

The -318CC genotype was decreased in the whole group of patients (p = 0.0003) and in the group with the mixed form (p<0.0001) compared to controls. A decreased frequency of the -318CC genotype was also observed in patients with the mixed form compared to digestive form (p<0.0001). The -318CT genotype frequency was increased in the group with the mixed form of the disease compared to controls (p<0.0001) and to patients with the cardiac (p = 0.0001) and digestive (p<0.0001) forms.

An increased frequency of the -318T allele was observed in the whole group (p<0.0001) and in the group with the mixed form (p = 0.0005) compared to controls. The -318C allele presented the opposite association pattern.

None of the genotype or allele frequencies of the CTLA-4 +49A/G polymorphism showed statistically significant difference between the different clinical forms of Chagas disease and healthy controls. The Odds Ratio and 95% Confidence Interval values obtained for the comparisons exhibiting significant differences are shown in Table 3. 

**Table 3:** Comparisons of genotype and allele frequencies of polymorphisms at the *CTLA-4* gene between different presentation forms of Chagas disease.

**Genotypes and Alleles**	**Comparison**	**OR(95%CI)**	**Comparison**	**OR(95%CI)**	**Comparison**	**OR(95%CI)**
**-1722CC**	IND *vs.* HC	4.51(2.1-9.5)	IND *vs.* CD	42.95(2.5-733.3)	IND *vs.* DG	36.69(2.2-627.4)
**-1722C**	IND *vs.* HC	3.48(2.2-5.5)	IND *vs.* CD	7.48(3.2-17.5)	IND *vs.* DG	7.45(3.0-18.4)
	IND *vs.* MX	7.69(2.3-26.0)				
**-1722T**	IND *vs.* HC	0.29(0.2-0.5)	IND *vs.* CD	0.13(0.05-0.3)	IND *vs.* DG	0.13(0.06-0.3)
	IND *vs.* MX	0.13(0.04-0.4)				
**-318CC**	WG *vs.* HC	0.53(0.4-0.7)	MX *vs.* HC	0.11(0.04-0.3)	MX *vs.* DG	0.11(0.04-0.4)
**-318CT**	MX *vs.* HC	7.33(3.0-18.2)	MX *vs.* CD	6.32(2.3-17.1)	MX *vs.* DG	11.93(4.2-33.6)
**-318T**	WG *vs.* HC	1.76(1.4-2.3)	MX *vs.* HC	2.58(1.6-4.3)		
**-318C**	WG *vs.* HC	0.57(0.4-0.7)	MX *vs.* HC	0.39(0.2-0.7)		

IND (Indeterminate). CD (Cardiac). DG (Digestive). MX (Mixed). WG (Whole Group). HC (Healthy Control). OR, odds ratio. 95%CI, 95% confidence interval. Statistically significant values at a 5% significance level after Bonferroni correction.

### Genotype and Allele Frequencies of *PD-1*.*3*G/A SNP

We also analyzed the association of an important polymorphism at the *PDCD1* gene in individuals infected with *T. cruzi*. Genotype and allele frequencies of the *PD-1*.*3*G/A polymorphism in patients with chronic Chagas disease and healthy controls are shown in Table 2. Neither the genotype nor allele frequencies of the *PD-1*.*3*G/A polymorphism showed statistically significant difference between the different clinical forms of Chagas disease and healthy controls.

The majority of SNP genotype distributions evaluated in this study adhered to the Hardy-Weinberg Equilibrium, only the *CTLA-4* -1722C/T SNP of the control group was not in equilibrium (p<0.05), due to an excess of the CC genotype.

### Haplotype frequency of polymorphisms at the CTLA-4 (-1722T/C; -318C/T; +49A/G) gene

To further understand how the ensemble of polymorphisms participates in the resistance/susceptibility to Chagas disease, we analyzed the haplotypes of SNPs observed at the *CTLA-4* gene. The Lewontin’s *D’* coefficient of Linkage Disequilibrium (LD) was evaluated between all three possible pairs of *CTLA-4* SNPs in both chagasic patients and healthy controls and failed to reveal significant LD only between SNPs -318 and +49 among chagasic patients (*D*’ = 0.1166; χ^2^ = 1.5825; p = 0.2084). Moreover, the likelihood ratio test of linkage disequilibrium indicated the existence of significant LD between all three possible pairs of *CTLA-4* SNPs (p = 0.0000 ± 0.0000 for each pair) in both groups (Table 4). Given the significant LD between all three possible pairs of *CTLA-4* SNPs, *CTLA-4* haplotype frequencies were estimated. Haplotype frequencies of polymorphisms at the *CTLA-4* gene of patients with chronic Chagas disease and controls are shown in Table 5. The CTLA-4 -1722T/-318C/+49G haplotype frequency was underrepresented in the whole group of patients (p<0.0001), as well as in the groups with the digestive (p = 0.0005) and indeterminate (p<0.0001) forms compared to controls.

**Table 4:** Linkage Disequilibrium standardized coefficient (*D*’) between *CTLA-4* SNPs among chagasic patients and healthy controls.

**Sample group (SNPs)**	***D*’**	**χ^2^**	**p-value**
HC (*CTLA-4* -1722 *vs. CTLA-4* -318)	0.8047	14.8505	0.0001
HC (*CTLA-4* -1722 *vs.* CTLA-4 +49)	0.5829	41.1311	0.0000
HC (*CTLA-4* -318 *vs.* CTLA-4 +49)	0.4739	26.6326	0.0000
WG (*CTLA-4* -1722 *vs. CTLA-4* -318)	0.8482	25.6487	0.0000
WG (*CTLA-4* -1722 *vs.* CTLA-4 +49)	0.3070	14.2043	0.0002
WG (*CTLA-4* -318 *vs.* CTLA-4 +49)	0.1166	1.5825	0.2084

HC (Healthy Control). WG (Whole Group).

**Table 5:** Haplotype frequencies of polymorphisms at the *CTLA-4* gene in patients with Chagas disease and healthy controls.

	**Clinical forms**											
**Haplotypes**	**IND**		**CD**		**DG**		**MX**		**WG**		**HC**	
	n	Freq.	n	Freq.	n	Freq.	n	Freq.	n	Freq.	n	Freq.
**-1722/-318/+49**												
TTG	11	0.079	17	0.112	10	0.078	6	0.083	44	0.089	29	0.046
CTA	0	0.000	0	0.000	0	0.000	1	0.014	1	0.002	2	0 0.003
TTA	44	0.314	40	0.263	30	0.234	26	0.361	140	0.285	131	0.210
CTG	0	0.000	1	0.007	0	0.000	1	0.014	2	0.004	1	0.002
TCG	12	0.086*	34	0.224	13	0.102*	8	0.111	67	0.136*	147	0.235
TCA	36	0.257*	55	0.361	69	0.539	29	0.403	189	0.384	260	0.415
CCG	16	0.114	5	0.033	2	0.016	1	0.014	24	0.049	42	0.067
CCA	21	0.150*	0	0.000	4	0.031	0	0.000	25	0.051	14	0.022

IND (Indeterminate). CD (Cardiac). DG (Digestive). MX (Mixed). WG (Whole Group). HC (Healthy Control). * frequencies that show statistical differences.

The indeterminate group exhibited a decreased frequency of the *CTLA-4* TCA haplotype (p = 0.0005) compared to controls. The *CTLA-4* TCA haplotype was also overrepresented in patients with the digestive form compared to the indeterminate (p<0.0001) group. An increased frequency of the *CTLA-4* CCA haplotype was observed in the indeterminate patients (p<0.0001) compared to controls, and also with patients presenting the cardiac (p<0.0001) and mixed (p = 0.0001) forms of the disease.

The Odds Ratio and 95% Confidence Interval values obtained for the comparisons exhibiting significant differences are shown in Table 6. Table 7 summarizes the major results obtained in this study.

**Table 6:** Comparisons of haplotype frequencies of -1722/-318/+49 polymorphisms at the *CTLA-4* gene between different presentation forms of Chagas disease.

**Haplotypes**	**Comparison**	**OR(95%CI)**	**Comparison**	**OR(95%CI)**
**TCG**	WG *vs.* HC	0.51(0.4-0.7)	DG *vs.* HC	0.37(0.2-0.7)
	IND *vs.* HC	0.31(0.2-0.6)		
**TCA**	IND *vs.* HC	0.49(0.3-0.7)	DG *vs.* IND	3.58(2.0-5.7)
**CCA**	IND *vs.* HC	7.71(3.8-15.6)	IND *vs.* CD	54.87(3.3-915.2)
	IND *vs.* MX	26.09(1.6-437.2)		

IND (Indeterminate). CD (Cardiac). DG (Digestive). MX (Mixed). WG (Whole Group). HC (Healthy Control). OR, odds ratio. 95%CI, 95% confidence interval. Statistically significant values at a 5% significance level after Bonferroni correction.

**Table 7:** Summary of the associations between genotypes, alleles and haplotypes of *CTLA-4* polymorphisms with Chagas disease and its clinical forms, showing increased or decreased frequencies of genotypes, alleles and haplotypes.

**Genotypes, alleles and haplotypes**	**Clinical forms**		
	**Indeterminate**	**Digestive**	**Mixed**
*CTLA-4* genotypes	↑ -1722CC		↓ -318CC
			↑ -318CT
*CTLA-4* alleles	↑ -1722C		↓ -318C
	↓ -1722T		↑ -318T
CTLA-4 -1722/-318/+49 haplotypes	↓ TCG	↓ TCG	
	↓ TCA		
	↑ CCA		

## Discussion

Chagas disease can be manifested under different clinical forms. In humans, the clinical pattern is tightly regulated by the magnitude of the immune response. Approximately 60% of patients remain asymptomatic throughout their lives after *T. cruzi* infection, characterizing the indeterminate form, probably by inducing a functional regulatory response accompanied by increased IL-10 production [37], which is able to efficiently control CD8^+^ T cells, promoting an anti-inflammatory milieu. These patients are also able to control the parasite burden after inducing cytotoxic NK cells [38]. On the other hand, Tregs play a pivotal role in the pathogenesis of Chagas disease, since Tregs from cardiac and digestive patients are not functional or are decreased in number and function, contributing to a pro-inflammatory microenvironment induced by IFN-γ-producing CD4^+^ T cells and TNF-producing monocytes [37]. In concert, these events promote parasite control and tissue damage [39]. 

CTLA-4 and PD-1 molecules are normally expressed on the Treg surface producing a negative signal and thus preventing T cell activation [40]. Polymorphic sites at genes encoding these regulatory molecules can unbalance the immune regulation during *T. cruzi* infection, altering Treg function and contributing to the appearance of distinct clinical forms in Chagas disease. In this study, we analyzed three polymorphic sites at the *CTLA-4* gene. Overall, the *CTLA-4* -1722 polymorphic site is less studied [41,42], whereas the *CTLA-4* -318 and CTLA-4 +49 polymorphic sites have been more frequently explored in several diseases [16,18,41-44]. We showed that the *CTLA-4* -1722CC genotype is strongly associated with the indeterminate form, i. e., this genotype is not observed in other clinical variants of Chagas disease, suggesting that the presence of this genotype protects against the development of the cardiac, digestive and mixed variants. Considering that this allele has been associated with an increased expression of CTLA-4 [19], it is possible that the increased expression of CTLA-4 on the Treg surface may ameliorate its function, controlling disease-induced damage. The absence of the *CTLA-4* -1722CC genotype in other Chagas disease variants may indicate that it can be a marker of the indeterminate form, suggesting that this genotype may protect against the development of the major chronic Chagas disease clinical manifestations. This finding supports the idea that patients exhibiting the indeterminate form have an increased immunoregulatory response.

Overall, the frequency of the *CTLA-4* -318T allele is increased in patients presenting the mixed form of the disease in relation to controls. In addition, the *CTLA-4* -318CT genotype is increased in the mixed form compared to cardiac and digestive groups as well as compared to controls, indicating that the *CTLA-4* -318T allele and the *CTLA-4* -318CT genotype are primarily associated with the mixed form of Chagas disease.

Besides the *CTLA-4* -1722 and *CTLA-4* -318 polymorphic sites that are located at the *CTLA-4* promoter region, we also investigated a polymorphic site located at position +49 in exon 1 of the *CTLA-4* gene. It was previously reported that the CTLA-4 +49AA genotype is associated with impaired T cell proliferation by increasing CTLA-4 function, whereas the CTLA-4 +49GG genotype impairs Treg function by increasing T cell proliferation and consequently causing a pro-inflammatory response [45]. However, no association was observed between the CTLA-4 +49 polymorphic site and the clinical forms of Chagas disease.

When we evaluated *CTLA-4* polymorphic sites as haplotypes (-1722T/C; -318C/T; +49A/G), we observed that the indeterminate patients are strongly associated with a reduced frequency of TCG and TCA haplotypes (probably associated with decreased expression/function of CTLA-4) and a higher frequency of CCA haplotype (probably associated with increased expression/function of CTLA-4), suggesting that these haplotypes confer an up-regulation of the CTLA-4 molecule. Thus, the expression of a specific *CTLA-4* haplotype can confer protection against or susceptibility to Chagas disease by modulating CTLA-4 function. It was previously shown that patients with the indeterminate form of the disease have a higher amount of IL-10-producing Treg cells [46] and increased number of CTLA-4^+^ CD8^+^ T cells compared to non-infected individuals and to cardiac patients [47].

Regarding PD-1, our group showed an up-regulation of PD-1 and its ligands on lymphocytes and APCs, respectively, after *T. cruzi* infection in murine models. After blocking PD-1 and its ligands, we observed a high inflammatory migration to the heart tissue, suggesting that this molecule is crucial to control inflammatory processes [48]. We also analyzed one polymorphic site at the *PDCD1* gene that encodes the PD-1 protein. Here, genotype or allele frequencies of the *PD-1*.*3*G/A polymorphism are not associated to the development of a specific clinical form of Chagas disease. Our data provide information of the importance of the *CTLA-4* gene as a genetic marker associated with different manifestation forms of chronic Chagas disease.

Taken together, our data show that polymorphic sites observed at genes encoding regulatory molecules might modulate the appearance of different clinical forms of Chagas disease. Overall, alleles, genotypes and haplotypes reported to increase the expression of the regulatory molecule CTLA-4 were associated with a milder form of the disease (indeterminate), suggesting that regulatory mechanisms play a crucial role during the course of Chagas disease.
